# Causes of the Gloss Transition Defect on High-Gloss Injection-Molded Surfaces

**DOI:** 10.3390/polym12092100

**Published:** 2020-09-15

**Authors:** Jinsu Gim, Eunsu Han, Byungohk Rhee, Walter Friesenbichler, Dieter P. Gruber

**Affiliations:** 1 Center for Coating Materials and Processing, Engineering Research Center, Seoul National University, 1, Gwanak-ro, Gwanak-gu, Seoul 08826, Korea; interactionjs@gmail.com; 2 Department of Mechanical Engineering, Ajou University, 206, Worldcup-ro, Suwon 16499, Korea; leunsul@gmail.com; 3 Department of Polymer Engineering and Science, Montanuniversität Leoben, A-8700 Leoben, Austria; walter.friesenbichler@unileoben.ac.at; 4 Polymer Competence Center Leoben GmbH, A-8700 Leoben, Austria; dieter.gruber@pccl.at

**Keywords:** gloss transition defect, surface defect, surface gloss, shrinkage, mold surface replication, surface analysis, injection molding

## Abstract

The gloss transition defect of injection-molded surfaces should be mitigated because it creates a poor impression of product quality. Conventional approaches for the suppression of the gloss transition defect employ a trial-and-error approach and additional equipment. The causes of the generation of a low-gloss polymer surface and the surface change during the molding process have not been systematically analyzed. This article proposes the causes of the generation of a low-gloss polymer surface and the occurrence of gloss transition according to the molding condition. The changes in the polymer surface and gloss were analyzed using gloss and topography measurements. The shrinkage of the polymer surface generates a rough topography and low glossiness. Replication to the smooth mold surface compensates for the effect of surface shrinkage and increases the surface gloss. The surface stiffness and melt pressure influence the degree of mold surface replication. The flow front speed and mold temperature are the main factors influencing the surface gloss because they affect the development rate of the melt pressure and the recovery rate of the surface stiffness. Therefore, the mold design and process condition should be optimized to enhance the uniformity of the flow front speed and mold temperature.

## 1. Introduction

**Figure 1:**
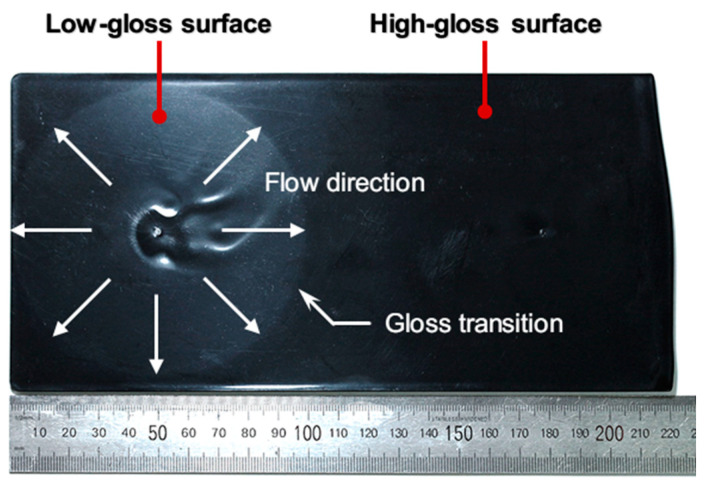
Example of the gloss defect on a high-gloss injection-molded surface.

A gloss transition defect is a visually recognizable transition on the surfaces of injection-molded products. The gloss transition defect is indicated by the gloss transition line between relatively low- and high-gloss areas perpendicular to the flow direction, as shown in Figure 1. It creates a poor impression of product quality. A low-gloss area in a high-gloss surface appears dusty and cloudy. A high-gloss area on a low-gloss surface looks like the area is worn out. The defect is readily visible under bright light conditions such as sunlight. Large exterior parts such as automobile trims are susceptible to the gloss transition defect. Post-processes such as coating, painting, and vapor deposition could be affected by a gloss transition defect due to the difference in surface characteristics. Therefore, the gloss transition defect should be mitigated in the injection molding process.

Molding conditions enhancing the mold surface replication have been recommended to eliminate surface defects and increase surface gloss. High mold temperature has been suggested as the main parameter influencing the surface gloss because the formation of a frozen layer is affected by the temperature condition [1,2,3,4,5,6,7,8,9,10,11]. High cavity or packing pressure [1,2,4,8,10,11] and injection speed [1,2,10,11] increase the surface gloss but have a less significant effect compared to the mold temperature [2,10]. Additionally, the melt temperature [2,4,6,8], screw rotation speed [6], flow length [5,7], and melt viscosity [8] influence the surface gloss. The effect of these molding parameters was analyzed for overall surface gloss and not the gloss transition on a single surface.

Several methodologies have been proposed to mitigate the gloss transition defect. For a hot runner system applied to the molding of large-area products, a position-controlled valve pin was proposed to reduce the drastic fluctuations of the molding condition [12,13]. Owing to the lack of optimization methodologies for the position profile of valve pins, a trial-and-error approach needs to be applied. A high mold temperature enhancing the surface gloss increases the cooling and cycle times. Thus, rapid heat and cool molding (RHCM) and variothermal techniques have been suggested to avoid the increase in cooling and cycle times [14,15,16,17]. A mold surface coating with an insulation film was proposed to replicate the effect of the high mold temperature without the RHCM controller [18,19].

The surface defects mainly depend on the molding condition in the filling stage. Yoshii [20,21] and Tredoux [22,23] proposed that wavelike flow marks are induced by the go-over phenomena and nonuniform thermal contraction under a low injection speed in the filling stage. Yokoi [24] and other researchers [25,26] proposed that the tiger stripes observed in the alternating gloss-dull stripes are generated at high injection speeds, and they are attributed to the flow instability related to the Weissenberg number in the filling stage. For the gloss transition defect generated in the middle range of injection speeds, Isayev and Kim pointed out that stable surface gloss can be achieved by constantly maintained melt flow [27]. Jeon et al. [28], Yuan et al. [12], and Bott [29] proposed that the hesitation of the flow causes the gloss defect and referred to it as flow hesitation or the halo mark. Suhartono et al. [13] showed that the stress distribution of the numerical simulation result near the hot gates resembles the gloss transition and referred to it as a stress mark. Yuan et al. [30] observed that the sudden fluctuation of the melt pressure causes the gloss transition defect, and referred to it as a pressure transition mark. Prior studies on the gloss transition defect proposed that enhanced mold surface replication increases the surface gloss. However, existing studies have not investigated the generation of a low-gloss polymer surface in a highly polished mold, as shown in Figure 1, and the influence of the filling condition on the surface gloss.

In this article, the causes of the gloss difference and the molding conditions for the mitigation of the resultant gloss transition defect are proposed. The surface characteristics determining the gloss difference are analyzed using surface topography and gloss measurements. The generation of a low-gloss polymer surface and the change in the polymer surface during the filling stage are analyzed using a short-shot specimen. The effect of the main process parameters on the surface gloss is investigated by analyzing the design of experiment (DOE) and the proposed causes. Consequently, the molding conditions for mitigating the gloss transition defect are proposed.

## 2. Materials and Methods

### 2.1. Materials

#### 2.1.1. Polymer Material

Poly (acrylonitrile-*co*-butadiene-*co*-styrene) (ABS) used herein is appropriate for the investigation of the gloss transition defect because its injection-molded surface is particularly sensitive to surface defects [1]. Furthermore, the black ABS HF380 (color code 9001) manufactured by LG Chem Ltd. (Seoul, Korea) was selected to recognize the gloss difference easily. The ABS was dried at 80 °C for 4 h to prevent bubble formation on the surface through the evaporation of moisture content.

#### 2.1.2. Injection Molds

**Figure 2:**
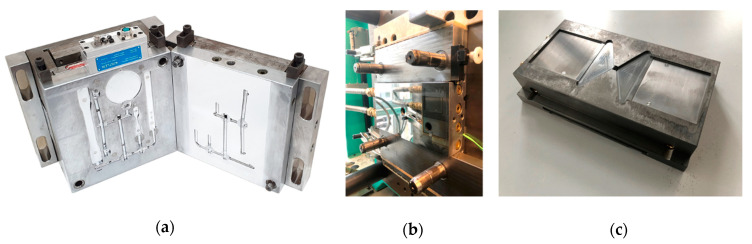
Injection molds: (**a**) mold type A, (**b**) fixed plate of the mold type B, and (**c**) moving plate of mold type B

Two different injection molds were used in this study. The first mold (mold type A) was used for ASTM specimens, as shown in Figure 2a. The gloss transition from the flow front in the filling stage appears along the long flow length of the short-shot specimen. A cavity with a long flow length was selected in the mold for the investigation of the polymer surface in the filling stage. The dimensions of the cavity were 12.7 mm × 127 mm × 3.2 mm. The dimensions of the gate were 6.3 mm × 3.2 mm. The air vent with the same width of the cavity was located at the end of the filling position. The other cavities in the mold were blocked at the runner branches. The areal root-mean-square (RMS) roughness of the mirror-polished mold surface was under 5 nm, and it was measured using the GPI XP/D interferometer of Zygo Corporation (Middlefield, CT, USA).

The second mold (mold type B) was used for the ISO D2 specimen, as shown in Figure 2b,c. It was selected for the analysis of the effect of the molding condition on the surface gloss. The dimensions of the cavity were 60 mm × 60 mm × 2 mm. It had a fan gate for obtaining a uniform flow pattern, as shown in Figure 2c. The dimensions of the gate were 60 mm × 1.5 mm. The surface of the fixed mold was mirror-polished, as shown in Figure 2b. At the surface of the fixed mold, the cavity pressure was measured using the Type 6190CA pressure sensor and the Type 5018 charge amplifier of Kistler AG (Winterthur, Switzerland).

### 2.2. Experiment Conditions

#### 2.2.1. Surface Changes in the Filling Stage

The change in the polymer surface in the filling stage needs to be investigated with respect to the gloss transition defect because a new polymer surface is generated, and it contacts the mold surface in the filling stage. The short-shot specimen represents the generation and change in the polymer surface during the filling stage. The high-speed electric injection-molding machine LGE150IIIDHS of LS Mtron Ltd. (Anyang, Korea) with a clamping force of 150 tons and the mold type A were used to mold the short-shot specimen. The flow front speed varies in the range of 10–100 mm/s for large area injection-molded parts such as TV back covers [31] and automotive bumpers. To investigate the change of the polymer surface in a wide range of filling conditions, the range of flow front speeds was set as 10–1000 mm/s. The jetting and burn marks were not generated in all experimental conditions because the mold type A had a large-area gate with the same thickness to the cavity and a wide air vent. The packing stage was not employed. Twenty cycles of the stabilization process were conducted before acquiring the specimen. The molding conditions are presented in Table 1.

#### 2.2.2. Influence of Process Parameters on Surface Gloss

**Figure 3:**
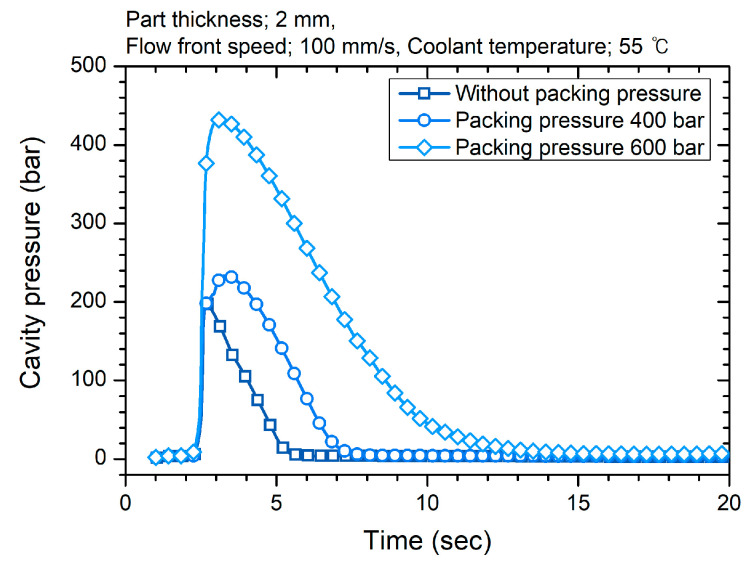
Cavity pressure under different packing pressure conditions.

Three process parameters were selected based on the factors expected to influence the surface gloss. The flow front speed in the filling stage is expected to be the main factor influencing the gloss transition defect. The packing pressure in the packing stage is predicted to enhance the surface gloss because it pressurizes the polymer surface to the mold surface. The mold temperature directly determined by the coolant temperature suppresses the gloss transition defect. The values of the coolant temperature were selected in the range of molding conditions recommended by the material manufacturer. The values of the flow front speed reflect the range of flow front speed for conventional injection molding conditions of large-area parts [31]. The minimum value of the packing pressure maintained the maximum cavity pressure at the end of filling, and the maximum value of the packing pressure doubled the cavity pressure, as shown in Figure 3. Selected process parameters and levels are listed in Table 2. The DOE was a full factorial design. The data analysis software Minitab 16.2 analyzed the effect of the process parameters on the surface gloss. The electric injection-molding machine Allrounder 470 A 1000-400 of Arburg GmbH (Lossburg, Germany) with a clamping force of 100 tons and the mold type B were used. The barrel temperature was set to 215 °C. Twenty cycles of the stabilization process were conducted before each DOE condition. The cavity pressure measurement showed that the packing time of 5 s was longer than the gate solidification time.

### 2.3. Measurement and Analysis

#### 2.3.1. Surface Gloss

The standard glossmeter GL0020 DuoGloss of TQC Sheen B.V. (Capelle aan den IJssel, Netherlands) was used to measure the surface gloss of the injection-molded surfaces. The geometry of the glossmeter complied with the standard test methods for surface gloss [32,33,34]. The surface gloss was quantified by the specular gloss value in gloss units (GU). The resolution of the glossmeter was 0.1 GU within the range of 0–100 GU. A measurement geometry of 20° was selected owing to the high glossiness of the specimen surface. The sizes of the measuring spot and detector aperture were 5 mm × 5 mm and 1.8° × 3.6°, respectively. A measuring pad with a black matt fabric material (0.0 GU for 20° geometry, 0.2 GU for 60° geometry) was used to eliminate the influence of surrounding light. The glossmeter was calibrated using the standard specimen included in the glossmeter set before the measurement of each specimen. For each specimen, a flat surface without a sink mark influencing the gloss measurement was measured.

#### 2.3.2. Gloss Distribution

**Figure 4:**
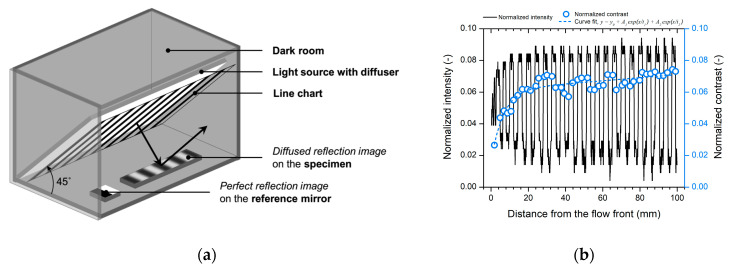
Gloss distribution measurement using the IPA technique [35,36]: (**a**) measurement setup and (**b**) intensity profile and contrast distribution.

The intensity profile analysis (IPA) developed by the Polymer Competence Center Leoben (PCCL) was employed to measure the gloss distribution on the short-shot specimen [35,36]. The measurement of the gloss distribution using the standard glossmeter requires repetitive measurements at different surface positions due to the small measuring area. The IPA based on the evaluation of the modulation transfer function can measure the gloss distribution close to human vision using a single measurement with high precision [35,36]. According to the visual test method of gloss difference [37], the contrast of the reflected image reveals the quality of the surface reflection and gloss. Figure 4a shows the measurement setup. The measurement was conducted in a dark room to prevent the influence of external lights. The line chart was illuminated by the diffused light source on the rear side. The high-contrast image of the line chart was projected onto the specimen surface and reference mirror. The reflected images on the specimen and mirror were both captured using the digital single-lens reflex camera EOS 700D with the EF-S 18–55 mm lens of Canon Inc. (Tokyo, Japan).The aperture was closed to F/9 to ensure a deep focal depth. The reflected image of the reference mirror was employed to focus on the line chart and normalize the intensity of the captured image. The positional contrast was derived from the intensity profile, as shown in Figure 4b.

#### 2.3.3. Surface Topography

The interferometer DCM8 of Leica Microsystems GmbH (Wetzlar, Germany) was used to measure the surface characteristics of the high-gloss polymer surface. Interferometry is a suitable measurement technique for a high-gloss polymer surface because it does not damage the low-hardness polymer surface, and has sufficient resolution (0.1–1 nm) for the analysis of the glossy surface, which has a lower roughness than the wavelength of visible light (380–740 nm) [38]. The surface was evaluated using a Mirau 50× lens in the phase-shifting interferometry mode with a green light source. The vertical resolution was 0.1 nm, and the lateral resolution was approximately 0.3 μm. The measured area was 351 μm × 261 μm.

**Figure 5:**
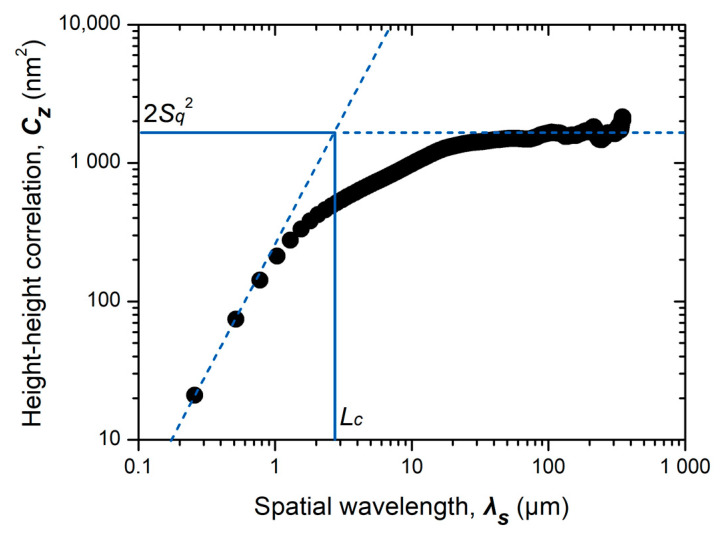
Determination of the root-mean-square (RMS) roughness (*S_q_*) and lateral correlation length (*L_c_*) using the height–height correlation function (HHCF).

The topography parameters, RMS roughness (*S_q_*), and lateral correlation length (*L_c_*) were determined by the height–height correlation function (HHCF, *C_z_*) of the measured surface, as shown in Figure 5. HHCF is defined as follows [39]:

*C_z_*(*λ_s_*) = [(*z*(*x* + *λ_s_*) − *z*(*x*))^2^] (1)

where *z* is the surface height, *x* is the lateral position, and *λ_s_* represents the spatial wavelength. The square bracket represents the average value of the term in the bracket for all lateral positions. The RMS roughness and lateral correlation length indicate the characteristic scales of the amplitude and wavelength of the surface fluctuation, respectively. The measured topography was analyzed using the open-source software for scanning probe microscopy, Gwyddion 2.52 [40].

## 3. Results and Discussion

### 3.1. Gloss Difference Induced by Surface Topography

**Figure 6:**
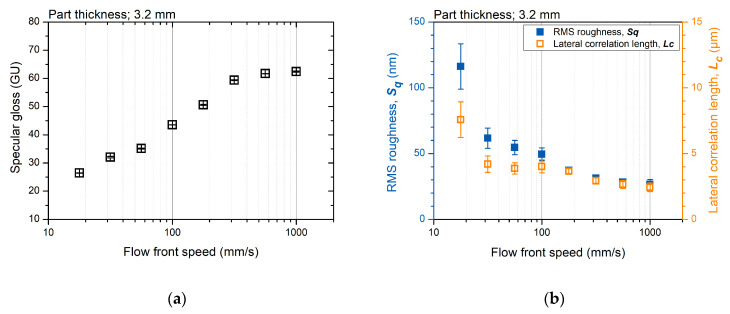
Surface measurement result: (**a**) surface gloss and (**b**) topography parameters.

The surface gloss and topography parameters show a similar tendency to the flow front speed. Figure 6a,b shows the measured surface gloss and the topography parameters of the specimen molded at various flow-front speeds, respectively. The surface gloss increased as the flow front speed increased. The RMS roughness and lateral correlation length decreased as the flow front speed increased. The surface gloss and topography parameters converged at a high flow-front speed. This similar trend for different molding conditions indicates a strong correlation between the surface topography and gloss.

**Figure 7:**
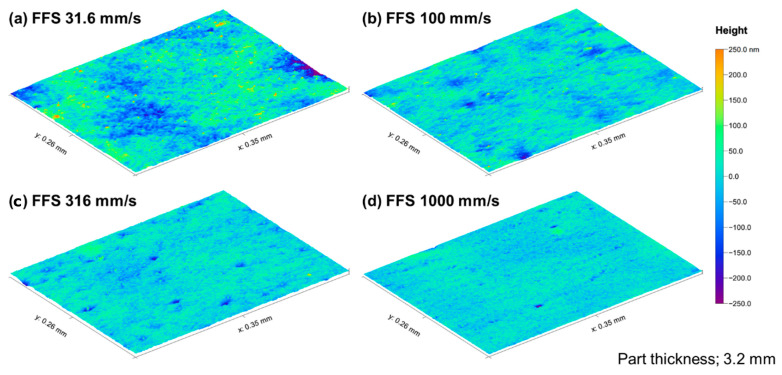
Surface topography molded by various flow-front speeds (FFS); (**a**) 31.6 mm/s, (**b**) 100 mm/s, (**c**) 316 mm/s, and (**d**) 1000 mm/s.

The theory of light-scattering phenomena can explain the relationship between surface topography and gloss. This is because the gloss depends on the degree of the reflected light concentration at the specular angle. As shown in Figure 7, the relatively low-gloss surface molded by a low flow-front speed shows a rough topography. This rough surface scatters the reflected light over a broad angular range. The rough surface induces diffused reflection and appears less glossy. The surface gloss can be modeled by the Kirchhoff theory of light scattering according to the surface roughness [41]. According to the modified Kirchhoff theory suggested by Alexander-Katz and Barrera [42], the surface gloss can be modeled as a function of the RMS roughness and lateral correlation length.

**Figure 8:**
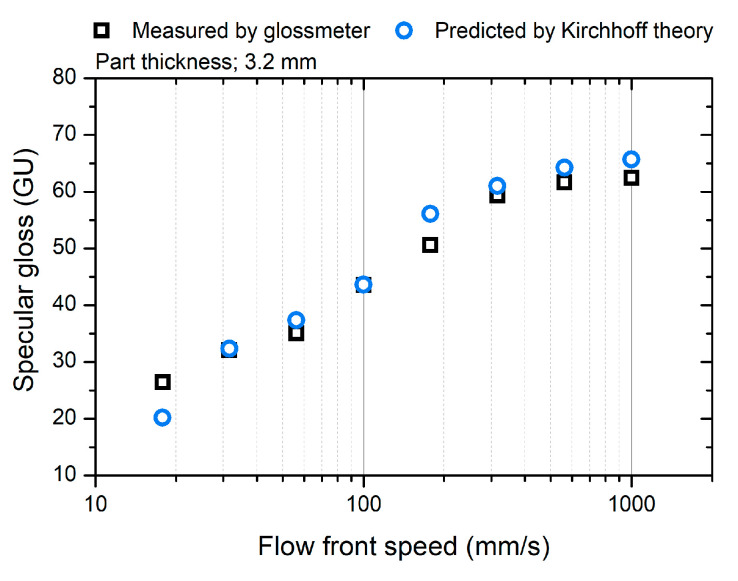
Comparison of the measured and predicted surface gloss.

Figure 8 shows a comparison of the measured and predicted surface gloss. The surface gloss was predicted using the Kirchhoff theory, measured topography parameters, the characteristics of the glossmeter [34], and the refractive index (1.515) of the ABS material [43]. The predicted surface gloss is consistent with the measured gloss. This indicates that the difference in the surface topography results in a gloss difference. The difference in surface gloss can represent the influence of the molding condition on the surface topography.

As shown in Figure 6b, the surface roughness determining the gloss difference is in a higher range (>20 nm) than that of the mold surface (<5 nm) and varies with the molding condition. The larger roughness of the polymer surface than that of the mold surface results from surface shrinkage. The decrease in the surface roughness is related to the effect of the molding conditions on the mold surface replication, as Oliveira et al. proposed [8]. The generation of a rough polymer surface and the mold surface replication occur in the filling stage.

### 3.2. Surface Changes in the Filling Stage

**Figure 9:**
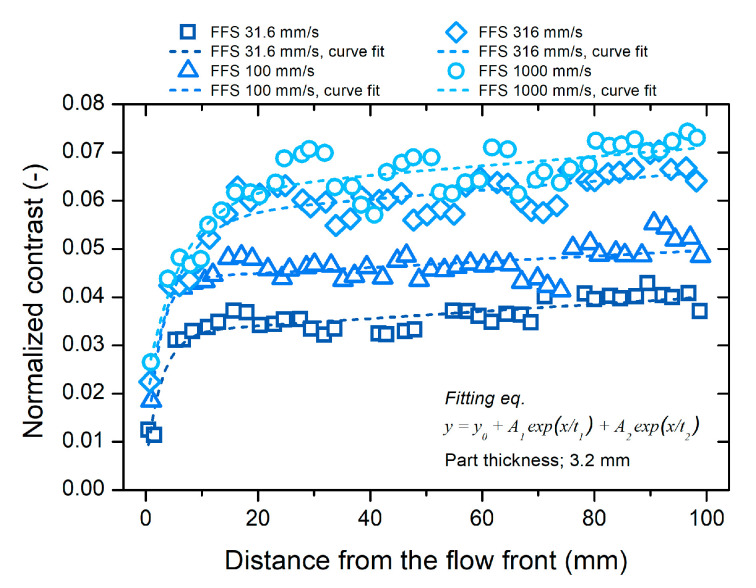
Contrast distribution of the short-shot specimen.

The gloss distribution of the short-shot specimen was analyzed to investigate the change in the polymer surface during the molding process. Figure 9 shows the distributions of the surface gloss on the short-shot specimen along the distance from the flow front. The contrast in Figure 9 indicates the surface gloss. As the distance from the flow front increased, the surface gloss increased. In particular, the gloss increased rapidly at a distance less than 20 mm, and gradually at a distance greater than 20 mm. A higher flow-front speed resulted in a more rapid increase in the surface gloss near the flow front. A similar distribution was observed in all the specimens molded in a wide range of flow front speeds. The distribution of the surface gloss could be explained by two factors: the generation of the rough surface due to surface shrinkage and the mold surface replication due to the melt pressure.

### 3.3. Rough Surface Generation

**Figure 10:**
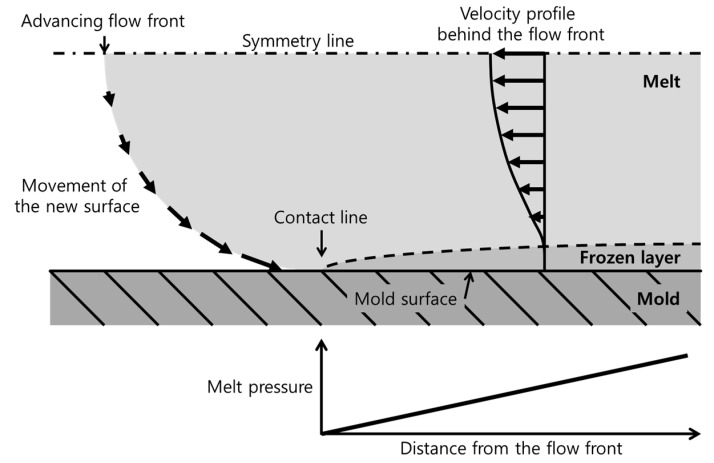
Fountain flow and melt pressure development at the flow front.

The rough polymer surface was generated after contact with the mold surface. During the filling stage, the fountain flow at the flow front generated a new polymer surface and transported the polymer surface to the mold surface, as shown in Figure 10. Then, the polymer surface touched the mold surface. The polymer surface was smooth owing to the elongational stress at the flow front. After contacting the mold surface, the polymer surface cooled down and shrunk.

The shrinkage crumples the polymer surface, resulting in a rough surface. Inhomogeneous shrinkage due to subsurface morphology affects the surface topography [44]. ABS contains butadiene rubber particles in the poly(styrene-*co*-acrylonitrile) (SAN) matrix. The size of the rubber particles ranges from 0.5 to 5 μm [45,46]. The rubber particles make the subsurface morphology complex and induce rough topography. Rosato reported a similar generation of rough surfaces due to the two phases in ABS [2]. Therefore, surface shrinkage generates rough and low-gloss polymer surfaces in highly polished injection molds.

**Figure 11:**
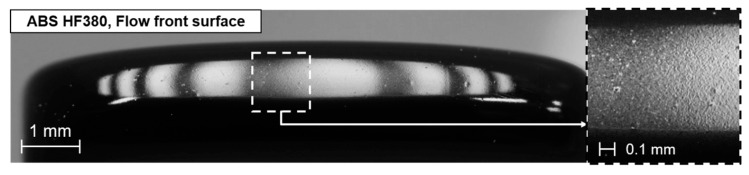
Rough surface at the flow front.

The generation of a rough surface by the surface shrinkage is maximized near the flow front of the short-shot specimen because the surface is not sufficiently pressurized by the melt pressure. If the filling stops before the cavity is fully filled, the generation of a new surface at the flow front stops. The polymer surface near the flow front freely shrinks because the distance near the flow front is too short to develop the melt pressure. Therefore, the surface at the flow front shows a rough topography. Figure 11 shows the rough surface at the flow front. The surface gloss near the flow front is at a minimum owing to the maximized surface shrinkage, as shown in Figure 9.

### 3.4. Mold Surface Replication

In Figure 9, the increasing gloss along the distance shows the effect of the melt pressure development on the surface topography. As the distance from the flow front increased, the melt pressure developed, as shown in Figure 10, and pushed the polymer surface to the smooth mold surface. The polymer surface contacting the mold surface shrunk simultaneously. A sufficient melt pressure enhanced the replication of the polymer surface to the smooth mold surface, and the surface shrinkage was compensated. The generation of a rough surface was due to surface shrinkage and mold surface replication due to the melt pressure occurring simultaneously. As the distance from the flow front increased, the generation of the rough surface was suppressed to a greater extent by a higher melt pressure. The surface gloss increased along the distance, as shown in Figure 9.

The rapid increase in the surface gloss near the flow front in Figure 9 was related to the stiffness recovery time of the polymer surface. The temperature of the polymer surface decreased from the melt temperature to the contact temperature immediately after contacting the mold surface. According to the heat conduction model between the melt and the mold suggested by Yoshii et al. [20], and Carslaw and Jaeger [47], the contact temperature was close to the mold temperature because the mold steel had a much higher thermal diffusivity than the polymer material. The subsurface temperature in the melt was higher than the contact temperature. As the contact time increased, the subsurface temperature converged to the contact temperature. Surface cooling recovered the surface stiffness. The recovery of the surface stiffness proceeded as the thickness of the frozen layer increased, as shown in Figure 10. The surface stiffness resisted the mold surface replication and the compensation of the shrinkage by the melt pressure. Until the surface stiffness recovered sufficiently to resist the melt pressure, the amount of mold surface replication increased rapidly. The surface gloss increased rapidly near the flow front, where the polymer surface was soft owing to the short cooling time.

The level of the surface gloss was determined in the region near the flow front. During the filling stage, the surface stiffness was recovered sufficiently far from the flow front. The melt pressure could not enhance the mold surface replication as much as near the flow front. The increasing rate of surface gloss reduced far from the flow front, as shown in Figure 9. This indicates that the difference in the surface gloss was determined near the flow front, and the filling stage dominantly affected the gloss transition defect. The effect of the molding conditions, including the filling condition, on the surface gloss was investigated via DOE analysis.

### 3.5. Influence of Molding Conditions on Surface Gloss

**Figure 12:**
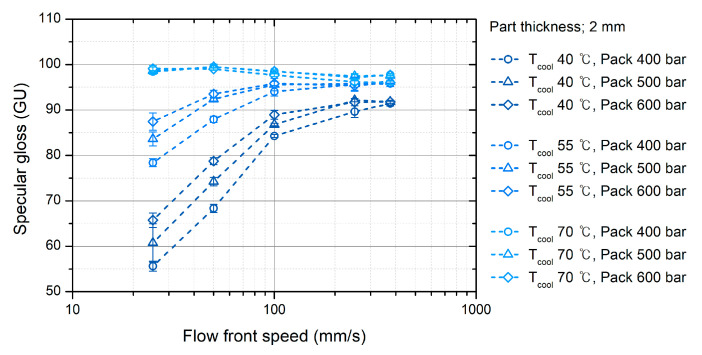
Surface gloss under various molding conditions.

**Figure 13:**
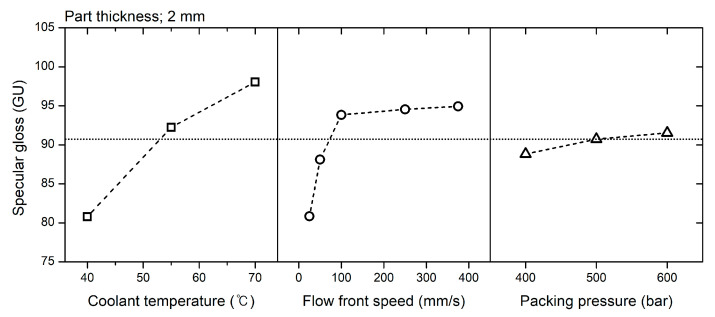
Effect of the molding conditions on the surface gloss.

Figure 12 shows the surface gloss of the specimens molded under various injection-molding conditions. The correlation between the molding conditions and the surface gloss was analyzed using DOE. Figure 13 shows the factorial plots representing the effect of the molding conditions on the surface gloss. Table 3 shows the analysis of variance (ANOVA) result. Coolant temperature and flow front speed were considered significant factors influencing specular gloss because *p*-values of these factors were under 0.05. Packing pressure was not a significant factor.

#### 3.5.1. Effect of Mold Temperature

The effect of the coolant temperature (*T_cool_*) on the surface gloss represents the influence of the mold temperature because the coolant temperature dominantly determines the mold temperature. The mold temperature had the greatest influence on the surface gloss, as shown in Figure 13. An increase in the mold temperature enhanced the overall surface gloss. This result agrees with the previous research [1,2,3,4,5,6,7,8,9,10,11]. At a high mold temperature (*T_cool_* of 70 °C), the surface shows the highest surface gloss value in Figure 12. The high mold temperature decreased the cooling rate of the polymer surface so that the temperature of the polymer surface was higher than that at a low mold temperature. The recovery rate of the surface stiffness and shrinking rate decreased. This indicates that the melt pressure pressurized the softer polymer surface over a longer time. The generation of the rough surface due to the shrinkage was suppressed, and the mold surface replication was enhanced at a high mold temperature.

An imbalance in the mold surface temperature can induce the gloss transition defect even if the mold surface was highly polished. Owing to the high thermal conductivity of the mold steel, the temperature did not change significantly over the mold surface in a single cycle. In the case of a mold with a poor thermal design, such as nonoptimized cooling channels with a high distance from each other and the cavity wall, the heat from the melt can be accumulated locally and the temperature deviation can be approximately 10 °C [48]. For example, the corner in the cavity is easily far from cooling channels and susceptible gloss defects [49]. It is desirable to maintain a uniform mold surface temperature to suppress the gloss transition defect. Optimization of the conventional cooling channel or conformal cooling channel is recommended in the mold design step.

The fluctuation of the surface gloss can be suppressed at high mold temperatures, as shown in Figure 12. The glass transition temperature of the ABS was 99 °C. As the mold temperature approaches the glass transition temperature of the polymer material, the recovery of the surface stiffness is considerably suppressed, and the polymer surface can replicate sufficiently to the mold surface even at a slow development of melt pressure. A high mold temperature is recommended when it is difficult to adjust the other molding conditions or to revise the mold design.

#### 3.5.2. Effect of the Flow Front Speed

The flow front speed in the filling stage is a significant parameter on the surface gloss as much as the mold temperature. The mold temperature has the largest influence on the surface gloss as Figure 13 and as reported by prior research [1,2,3,4,5,6,7,8,9,10,11]. In comparison with the mold temperature inducing a gradual increase of the surface gloss, the flow front speed increased rapidly the surface gloss below 100 mm/s of the flow front speeds, as shown in Figure 13.

The flow front speed influenced the increasing rate of the melt pressure. The distance from the flow front and the flow front speed increased the melt pressure development at a specific location on the mold surface [50]. As the flow front speed increased, a higher melt pressure pressurized the soft polymer surface until the surface stiffness recovered sufficiently. The flow front speed mainly influenced the surface near the flow front during the filling stage, as shown in Figure 8.

The increment in the surface gloss due to the flow front speed converged at a high flow-front speed. For example, the surface gloss sharply increased at the coolant temperature of 40 °C and flow front speeds less than 100 mm/s, as shown in Figure 12. However, the surface gloss converged to approximately 90 GU at flow front speeds greater than 100 mm/s. This is due to the difference in the rates of surface cooling and pressure development. If the flow front speed is sufficiently high, the development of the melt pressure is faster than the recovery of the surface stiffness. A sufficiently high melt pressure maximizes the mold surface replication of the polymer surface. The fluctuation of the surface gloss due to the flow front speed stabilizes at high flow-front speeds. This indicates that the gloss transition defect can be suppressed by a high flow-front speed.

**Figure 14:**
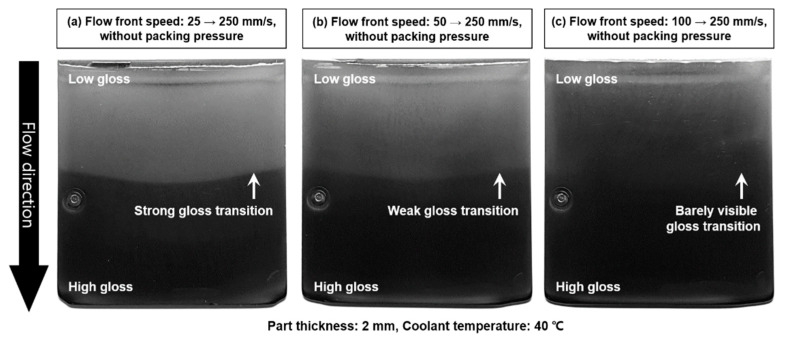
Effect of the flow front speed fluctuation on the gloss transition defect; (**a**) flow front speed fluctuation 25–250 mm/s, (**b**) flow front speed fluctuation 50–250 mm/s, and (**c**) flow front speed fluctuation 100–250 mm/s.

The fluctuation of the flow front speed can induce the gloss transition defect. The flow front speed may fluctuate in the filling stage and induce the gloss transition defect, as shown in Figure 14. The flow front speed increased to 250 mm/s at the middle of the surface and induced the strong gloss transition defect, as shown in Figure 14a. The fluctuation of the flow front speed in a high flow-front speed range decreased the degree of the gloss transition defect. The flow front speed was changed from 100 to 250 mm/s at the middle of the specimen in Figure 14c and made barely visible the gloss transition. This result represents that the gloss transition defect can be suppressed in a high flow-front speed range even if the flow front speed fluctuates in the filling stage.

The flow pattern according to the cavity geometry affects the distribution of the flow front speed. For example, the flow front speed gradually decreased in a radial flow pattern. In addition, the operation of the injection-molding machine and hot runner system influenced the fluctuation of the flow front speed. For example, the sequential operation of the valve gate in the hot runner system caused significant fluctuations in the flow front speed near the valve gate owing to the sudden release of a high pressure and melt compression in the manifold. Therefore, the mold design and the process parameters related to the flow front speed should be optimized to minimize the fluctuation of the flow front speed.

#### 3.5.3. Effect of Packing Pressure

**Figure 15:**
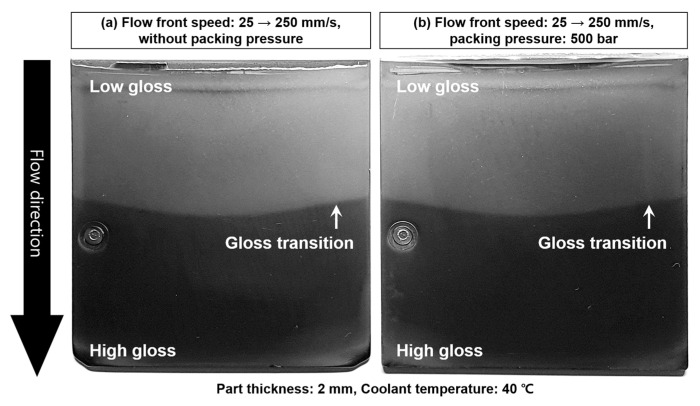
Effect of the packing pressure on the gloss transition defect caused by the flow fluctuation 25–250 mm/s; (**a**) without packing pressure, and (**b**) with 500 bar of packing pressure.

The packing pressure increased the surface gloss, as shown in Figure 12. The pressure that pushed the polymer surface to the mold surface enhanced the surface gloss. In the filling stage, the melt pressure pressurized the polymer surface. After the filling stage, the packing pressure additionally pressurized the polymer surface. The influence of the packing pressure on the surface gloss was not as high as that of the flow front speed and mold temperature, as shown in Figure 13 and this result is in agreement with the report proposed by Posciotti et al. [11]. The packing pressure could not eliminate the gloss transition defect already generated in the filling stage, as shown in Figure 15. This is because the packing pressure was applied to the already stiffened polymer surface. The surface stiffness was already recovered when the packing stage started. The effect of the packing pressure was similar to the slow increase of the surface gloss due to the melt pressure far from the flow front, as shown in Figure 9. This result indicates that the packing pressure cannot eliminate the gloss difference already created in the filling stage.

## 4. Conclusions

In this study, the causes of the gloss transition defect were investigated via surface topography and gloss measurements. The generation of a low-gloss surface and the change in the surface during the molding process were analyzed to determine the causes of the gloss transition defect. The effects of the molding conditions on the surface gloss were described. Consequently, the molding conditions suppressing the gloss transition defect were proposed.

A low-gloss polymer surface can be molded even if the mold surface is highly polished. This is due to the generation of a rough polymer surface due to surface shrinkage. For polymer materials with complex morphologies such as ABS containing rubber particles, inhomogeneous shrinkage due to the complex morphology made the polymer surface rough. The rough surface induced diffused reflection, resulting in low glossiness. The mold surface replication compensated for the effect of the surface shrinkage and increased the surface gloss. The melt pressure and surface stiffness influenced the degree of mold surface replication. The melt pressure pushing the polymer surface to the mold surface enhanced the replication and generated high surface gloss. The surface stiffness resisted the melt pressure and mold surface replication. Therefore, the surface shrinkage and the difference in the mold surface replication caused the gloss transition defect.

The filling condition mainly determines the surface gloss. The mold temperature and flow front speed were the parameters having the greatest influence on the surface gloss. The mold temperature influenced the recovery rate of the surface stiffness. At a high mold temperature, the polymer surface was soft for a longer time. The softer polymer surface could better replicate the mold surface. The flow front speed affected the development rate of the melt pressure, pushing the polymer surface to the mold surface. At a high flow front speed, rapidly increasing melt pressure pressurized the polymer surface and replicated the mold surface better. Therefore, the fluctuation of the flow front speed and the nonuniformity of the mold temperature in the filling stage generated the gloss transition defect.

The nonuniformity of the mold temperature and the fluctuation of the flow front speed should be minimized in the filling stage to suppress the gloss transition defect. The thermal design of the mold, such as the cooling channels, should be optimized to minimize the temperature deviation of the mold surface. It is desirable to optimize the operation of the injection unit of the molding machine and sequential valve gates to reduce the fluctuation of the flow front speed.

## Figures and Tables

**Table 1:** Injection-molding conditions for the short-shot specimen.

Process Parameter	Values
Coolant temperature (°C)	35
Barrel temperature (°C)	210
Flow front speed, FFS (mm/s)	17.8, 31.6, 56.2, 100, 178, 316, 562, 1000

**Table 2:** Process parameters for the design of experiment (DOE).

Process Parameter	Levels	Values
Flow front speed, FFS (mm/s)	5	25, 50, 100, 250, 375
Packing pressure (bar)	3	400, 500, 600
Coolant temperature (°C)	3	40, 55, 70

**Table 3:** Analysis of variance (ANOVA) for specular gloss.

Design Parameters	Degree of Freedom	Sum of Squares	Mean Square	F-Ratio	*p*-Value
Coolant temperature	2	3552.56	1776.28	45.77	0.000
Flow front speed	4	2007.25	501.81	12.93	0.000
Packing pressure	3	204.81	68.27	1.76	0.167
Error	50	1940.26	38.81		
Total	59	7704.89			
